# Ancoats Hospital and Amateur Actors

**Published:** 1913-12-20

**Authors:** 


ANCOATS HOSPITAL AND AMATEUR ACTORS.

The North Salford Amateur Dramatic Society have kindly given the opera "Iolanthe" for four evenings in aid of Ancoats Hospital, Manchester. A successful performance took place at the Cheetham Public Hall. Sisters White and Hayward, with two nurses from Ancoats Hospital, all in uniform, sold programmes, and picture postcards of the hospital were disposed of during the interval. We hope the result will mean a good sum being handed to the treasurer of the hospital.

